# Development of 12 Microsatellite Markers in *Dorcus titanus castanicolor* (Motschulsky, 1861) (Lucanidae, Coleoptera) from Korea Using Next-Generation Sequencing

**DOI:** 10.3390/ijms17101621

**Published:** 2016-09-23

**Authors:** Tae Hwa Kang, Sang Hoon Han, Sun Jae Park

**Affiliations:** 1 Animal Resources Division, National Institute of Biological Resources, 42, Hwangyeong-ro, Seo-gu, Incheon 22689, Korea; colkth@daum.net; 2 Department of Life Science, College of Natural Science, Kyonggi University, 154-42, Gwanggyosan-ro, Yeongtong-gu, Suwon 16227, Korea; 3 Biological Resources Research Department, National Institute of Biological Resources, 42, Hwangyeong-ro, Seo-gu, Incheon 22689, Korea

**Keywords:** *Dorcus titanus castanicolor*, microsatellite marker, HiSeq, next-generation sequencing, Lucanidae, Coleoptera, Korea

## Abstract

In the present study, we used next-generation sequencing to develop 12 novel microsatellite markers for genetic structural analysis of *Dorcus titanus castanicolor* (Lucanidae; Coleoptera), a popular pet insect in China, Korea, and Japan. We identified 52,357 microsatellite loci in 339,287,381 bp of genomic sequence and selected 19 of the loci based on their PCR amplification efficiency and polymorphism. The 19 selected markers were then tested for the presence of null alleles and linkage disequilibrium. We did not detect any evidence of null alleles; however, four pairs of loci (DT03 and DT11, DT05 and DT26, DT08 and DT26, DT26 and DT35) exhibited linkage disequilibrium. Thus, we assessed the genetic diversity of a *D. titanus castanicolor* population from the Daejeon region of Korea (*n* = 22) using 13 markers. Among them, one marker (DT17) deviated from Hardy-Weinberg equilibrium. Therefore, 12 markers may be useful for further analyzing the genetic diversity of *D. titanus castanicolor*.

## 1. Introduction

**Figure 1:**
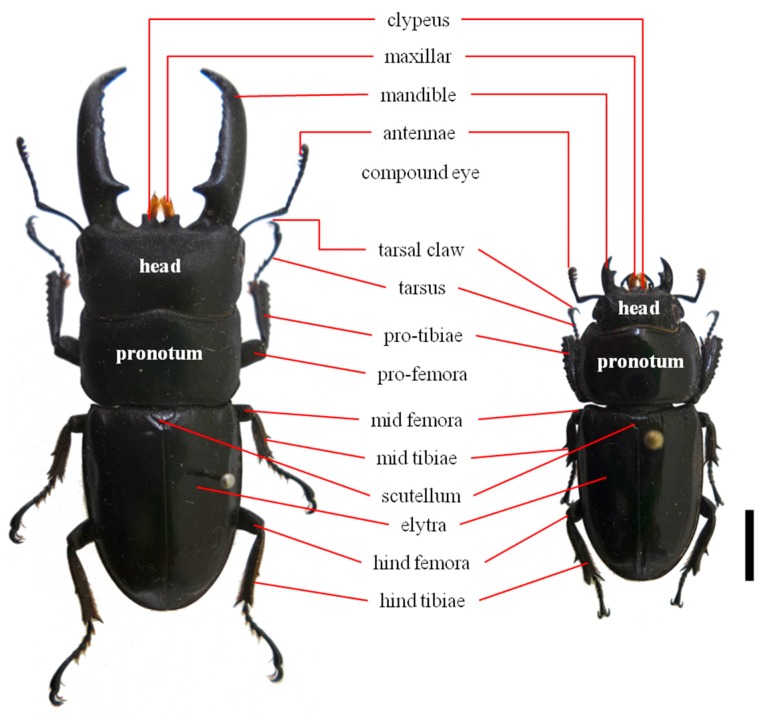
Adult photos and terminology of *Dorcus titanus castanicolor*—**left**, male; **right**, female (Scale bar = 1.0 cm).

*Dorcus titanus castanicolor* [1] is a subspecies of stag beetle that is distributed in northeast China, Korea, and Tsushima Island, Japan [2,3,4] and is distinguished from the other subspecies by a distinctly bilobed clypeus and flattened last abdominal sternite in males and moderately punctured elytra and straight protibiae in females [4] (Figure 1). In Korea, the subspecies is distributed throughout the country and is commonly found during local fauna and monitoring surveys [3,4].

Marketing campaigns from the insect industry have contributed to a rapid increase in the number of pet insects [5,6,7]. *D. titanus castanicolor* is a popular pet insect [7], and the rearing method of the subspecies is well documented [8]. In China and Japan, many stag beetle breeders have reared large-sized individuals of *D. titanus* [9]. Japan has 12 subspecies of *D. titanus*, which were interbred to select for large individuals. Consequently, four foreign subspecies (*D. titanus platymelus*, *D. titanus pilifer*, *D. titanus palawanicus*, and *D. titanus titanus*) were introduced to Japan without prior ecological assessment. Thus, ecological problems, such as decreases in the populations of native subspecies, have occurred, owing to competition with the introduced subspecies [9].

Recently, several Southeast Asian subspecies, such as *D. titanus palawanicus*, were introduced into Korea. Kim and Kim [4] conducted a comparative analysis of the male genitalia through a taxonomic review of the Korean Lucanidae and reported that the Korean subspecies (*D. titanus castanicolor*) might actually be a distinct species. A different case was reported for *Dorcus hopei* [10]. Thus, seven microsatellite markers were developed in order to help conserve the Korean population and used to compare the genetic structure of the Korean *Dorcus hopei* with other regional populations [11]. Recently, the Korean Quarantine Agency also used the markers for a quarantine inspection of foreign *Dorcus hopei*. Therefore, the goal of this study was to develop microsatellite markers for *D. titanus castanicolor* that could be used for population genetic analysis and origin testing.

## 2. Results and Discussion

### 2.1. Next-Generation Sequencing and Microsatellite Loci Identification

Through Illumina sequencing of *D. titanus castanicolor* genomic DNA, the number of contigs with length >1000 bp was 123,291. The maximum length was 103,231 bp, and the mean length was 2729 bp. We obtained 339,287,381 bp of genomic DNA sequence with a GC content of 38.98% (Table 1).

**Figure 2:**
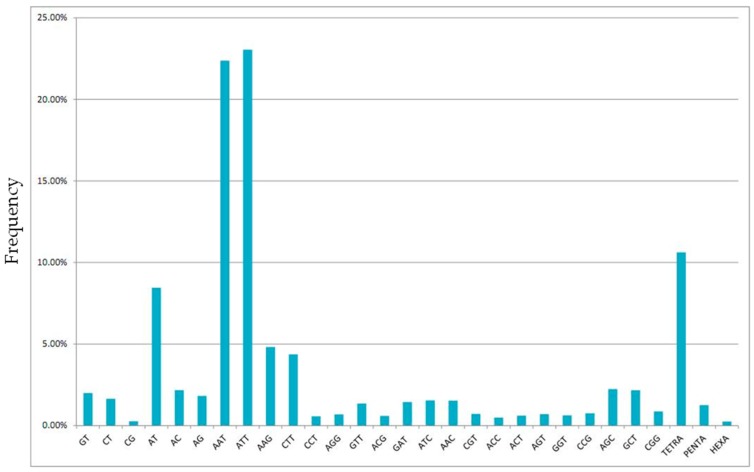
Frequency of short tandem repeats identified in the *Dorcus titanus castanicolor* genomic DNA sequence.

Searching the genomic DNA sequence yielded 52,357 microsatellite loci with 14,644 di-, tri-, and tetra-repeat sequence motifs, 186 penta-repeat loci, and 37 hexa-repeat loci. The relative frequencies of the specific repeated sequences were as follows: ATT, 24%; AAT, 23%; AT, 8%; and remaining motifs, below 5% (Figure 2).

The 14,644 searched microsatellite loci were filtered with two conditions. First, the loci composed of AT repeat were excluded because of difficulty in PCR. Second, the loci composed of 24–96 bases were selected. Using these two filtering conditions, we selected 316 microsatellite loci, and using the primer design software PRIMER3 version 0.4.0 [12,13], we designed primer sets for 301 loci. Eighty-five markers were selected based on the GC contents of the product sequences and melting temperatures of the designed primer sets and were subsequently tested for specificity and polymorphism. Finally, 19 markers were selected and the forward primers of each selected loci were labeled with a fluorescent dye (FAM) at the 5′ end [14] (Table 2).

### 2.2. Microsatellite Marker Assessment

The genetic diversity of the Korean *D. titanus castanicolor* (*n* = 22) was assessed, using the 19 selected markers. We determined the genotypes of 22 individuals using a 3730XL DNA analyzer (Applied Biosystems^®^, Foster City, CA, USA) (Table S1). Each marker was tested for PCR errors and the presence of null alleles (Table 3). Then, the linkage disequilibrium analysis was conducted for each locus. From these results, linkage disequilibrium was detected in four pairs of loci (DT03 and DT11, DT05 and DT26, DT08 and DT26, and DT08 and DT35), which were excluded from further analysis. Thus, we considered that the 13 remaining microsatellite loci might be suitable for analyzing the population genetic structure of Korean *D. titanus castanicolor*.

### 2.3. Genetic Diversity of the Korean Dorcus titanus castanicolor Population

Genetic diversity metrics for the 13 selected markers were calculated using PowerMarker version 3.25 [15] (Table 4). The genotype number, allele number, expected heterozygosity, and observed heterozygosity ranged from 3–11, 3–9, 0.3140–0.7789, and 0.3636–1.0000, respectively. Significant departure of genotype frequencies from Hardy-Weinberg equilibrium (HWE) was determined after sequential Bonferroni corrections for the significance level (critical *p* = 0.003). We found that only one locus (DT17) significantly deviated from HWE (Table 4). Increasing the benefit as a pet insect, the wild population of the Korean *D. titanus castanicolor* is decreasing because of over-collecting by the Korean breeders. Some Korean breeders surreptitiously made crosses between the Korean and exotic subspecies (such as *D. titanus palawanicus*) for body size and mandible morphology, like in Japan [9]. For these reasons, we anticipated that there could be multiple non-HWE markers. Although our data did not suggest this, further surveys are necessary using the 12 markers and additional populations of Korean *D. titanus castanicolor*.

## 3. Materials and Methods

### 3.1. Sample Collection and Genomic DNA Extraction

We collected 23 *Dorcus titanus castanicolor* specimens from Mt. Jangtae, which is located in Jangan-dong, Seo-gu, Daejeon, Korea (Table S2). We extracted the thoracic muscles of the specimens for genomic DNA isolation and deposited the dried samples in the National Institute of Biological Resources (Incheon, Korea). Genomic DNA for microsatellite loci amplification and genotyping was isolated using a DNA purification kit (Prime Prep Genomic DNA Isolation Kit, GeNet Bio, Daejeon, Korea) according to the manufacturer’s instructions. Genomic DNA for next-generation sequencing was extracted from one individual using the NucleoSpin^®^ Tissue Kit (Macherey-Nagel GmbH and Co. KG, Düren, Germany), and its quality was checked, using both spectrophotometry and electrophoresis on a 1% agarose gel.

### 3.2. Next-Generation Sequencing, Microsatellite Loci Identification, Marker Selection, and Genotyping

For next-generation sequencing, 10 μg of genomic DNA from a single specimen was used for 2 × 300 paired-end sequencing with a HiSeq Sequencer (Illumina, San Diego, CA, USA). Assembly was performed using the de Bruijn graph algorithm in CLC Genomics Workbench version 7 (CLC Bio, Aarhus, Denmark). Assembly mapping was performed after removing Illumina adapters and low-quality sequences using the CLC trimmer function (default limit = 0.05). The assembly procedure used the parameters Length Fraction (LF) and Sequence Similarity (SIM) between DNA reads, as described by the CLC Genomics Workbench software, with maximum stringency (0.50 LF and 0.80 SIM) and a minimum contig length of 70 bp.

Microsatellite loci were identified in the assembled partial draft genome using tandem repeat search software, Phobos version 3.3.12 [16,17]. The searching criteria were 10% mismatch di-, tri-, and tetra-nucleotide repeats with a sequence length between 24 and 96 bp, and AT repeats were excluded from the screening results because of difficulty in PCR. Primer sets for amplifying of the microsatellite loci were designed using PRIMER3 version 0.4.0 [12,13] with the following criteria: GC content >30%, primer length 18–22 bp, optimal temperature of 56–60 °C, and default settings for the remaining parameters. PCR for the qualification test on the designed primer sets was conducted in 20 μL reactions using AccuPower PCR PreMix (Bioneer, Daejeon, Korea), 30 ng template DNA, and 10 pmol each primer. Extra MgCl_2_ was not added. PCR amplification was carried out in a Veriti 96-Well Thermal Cycler (Applied Biosystems^®^, Foster City, CA, USA), using the following program: an initial denaturation step of 5 min at 94 °C; followed by 35 cycles of 10 s at 94 °C, 10 s at 56 or 60 °C, and 20 s at 72 °C; and a final extension step of 5 min at 72 °C. The specificity of the primer sets and polymorphism of the microsatellite loci were examined through electrophoresis of the PCR amplicon using QIAxcel (Qiagen, Leipzig, Germany). Each forward primer of the selected markers was labeled with 6-carboxyfluorescein at the 5′ end for genotyping [14]. To reduce polymerization error, high fidelity polymerase (SuPrime HF Premix (2×), *GeNet* Bio, Daejeon, Korea) was used in PCR for genotyping, which was carried out using the same conditions as those for the qualification test of the designed primer sets. The sequences of microsatellite loci for the developed markers were submitted to NCBI GenBank (Table 3).

### 3.3. Data Analysis

The presence of null alleles and PCR errors in the genotyping results was checked using Micro-Checker version 2.2.3 [18], and pairwise linkage disequilibrium was analyzed using Arlequin version 3.1 [19]. The genetic diversity of the Daejeon population was estimated using the major allele frequency (MAF), number of Genotypes (Gn), sample size (SS), number of alleles (An), expected heterozygosity (He), observed heterozygosity (Ho), polymorphism information content (PIC), and Hardy-Weinberg equilibrium (HWE), all of which were calculated using PowerMarker version 3.25 [15]. Deviation from HWE was determined after sequential Bonferroni correction for the significance level.

## Figures and Tables

**Table 1:** Summary of next-generation sequencing results from *Dorcus titanus castanicolor* genomic DNA.

Number of Contigs (>1000 bp)	Total Bases	Maximum Length	N50 Length	Average Contig Length	GC Content
124,291	339,287,381	103,231	3258	2729	33.98%

**Table 2:** Microsatellite markers selected for genotyping the 22 *Dorcus titanus castanicolor* specimens from Daejeon, Korea.

MSL	Forward Sequence	Reverse Sequence	RM	SS	MN	NAn
31,870	CGACACTCACACGCAATAAATAG	GCCATTCCATCGGTGAATTTTTA	(CGTT)_6_	214 bp	DT01	KX268665
13,508	TGAAGTTTCAGAAGGAGTTTGGA	CAATCAACTCCGGATAGAGTGAA	(ACAT)_6_	264 bp	DT02	KX268666
78,687	AGAACGCGTGAACTTGTTTATTT	TTGTCACGTTCTAAAGCGTTTTT	(AAG)_8_	244 bp	DT03	KX268667
9989	CCCCACGAACAATAAATCAGAAC	TACTTCCGCTTACAGCAGTAAAT	(CGT)_8_	202 bp	DT05	KX268668
9645	TTATGACGAACTTGTTTTGGCAG	TGTGGAATTTTGTTTCGGATGTT	(AC)_12_	298 bp	DT06	KX268669
8497	GTCATCTTACTAGAGGTTGGTCG	CAACACTTCTTCTAATGCACTCG	(AAG)_17_	205 bp	DT08	KX268670
15,216	ATTACGTAGACATGGCTTAAGCA	TCCTCATGCCAAATATGATCCAA	(AACT)_10_	245 bp	DT11	KX268671
29,993	TAATGACGCCATTCACACATTTC	GGTCTTTAAACAGTCTGCCAAAA	(GT)_20_	239 bp	DT12	KX268672
12,743	ACTTGACTGTCACATACACACAT	TTGTATGTCTGGCTTTGGTTACT	(CT)_19_	212 bp	DT13	KX268673
30,799	AAGACGATCGGCAATTTCAAAAT	TAGACCAGAGTCCTTCATTACCA	(AG)_17_	209 bp	DT15	KX268675
24,714	GAGCACCTAGATAATCAACCCAA	GATTTGGTTTCTTGCATTGTCCT	(AAG)_11_	230 bp	DT17	KX268676
3144	TCATTTGGACGTAGCAAATGTTC	TGCATGTTGAAAGGTACCAAGTA	(AAC)_10_	260 bp	DT24	KX675371
3488	TAATCCTTGCATGCAGCCTAATA	AGTTTGTGTTTGTGATGAAGTGG	(AGC)_10_	298 bp	DT25	KX675372
74,360	GTTATGGTGTGAGCTGGAATTTC	ATTTACATCAAGATTCGCCGTTG	(GTT)_10_	271 bp	DT26	KX675373
557	AACAAATAACGTGTTGGAGTTCG	AGTAGATGAGAACTGTGTTTGGG	(ACAT)_7_	284 bp	DT27	KX675374
4515	TCCACGTTATCTTTCAAAACACG	TTCAAGTTTGGGTGAAACAATCG	(CT)_14_	226 bp	DT28	KX675375
2260	AACATGACCCACTAAGGTATCAA	CTAACAAGAAGTACCACCACCAT	(GCT)_9_	300 bp	DT32	KX675376
16,503	ACGCTACTGGCAATCAAATAAAC	GCACCCACAAACAACATACATAA	(ACT)_9_	284 bp	DT33	KX675377
426	GCACGTCACGACTATTTTGATTT	TAACTCGTTAATGAACATGTGCG	(AC)_13_	210 bp	DT35	KX675378

MSL, microsatellite loci; RM, repeat motif; SS, size standard; MN, marker name; NAn, NCBI accession number.

**Table 3:** Statistical summary of the results from PCR errors and the null allele test in the Korean population of *Dorcus titanus castanicolor*.

**MSL**	**DT1**	**DT2**	**DT3**	**DT5**	**DT6**	**DT8**	**DT11**	**DT12**	**DT13**	**DT15**
NP	no	no	no	no	no	no	no	no	no	no
NFreq	−0.1909	−0.1408	−0.3352	−0.3970	−0.1199	−0.0986	−0.1269	−0.0874	−0.2445	−0.0930
**MSL**	**DT17**	**DT24**	**DT25**	**DT26**	**DT27**	**DT28**	**DT32**	**DT33**	**DT35**	–
NP	no	no	no	no	no	no	no	no	no	–
NFreq	−0.1109	0.0250	−0.0022	−0.6180	−0.0642	−0.0800	0.0171	−0.0144	−0.1185	–

MSL, microsatellite loci; NP, null allele present; NFreq, frequency of null allele.

**Table 4:** Characteristics of the 13 polymorphic microsatellite loci in the *Dorcus titanus castanicolor* specimens from Daejeon, Korea.

MSL	MAF	Gn	SS	An	He	Ho	PIC	HWE
DT1	0.8182	3	22	3	0.3140	0.3636	0.2918	1.0000
DT2	0.6591	5	22	6	0.5103	0.6364	0.4619	0.1100
DT6	0.3409	9	22	6	0.7789	0.9545	0.7466	0.0120
DT12	0.3409	10	22	6	0.7397	0.8636	0.6968	0.5770
DT13	0.3864	7	22	6	0.7118	1.0000	0.6647	0.0190
DT15	0.3636	9	22	7	0.7717	0.9091	0.7404	0.0510
DT17	0.4091	8	22	9	0.7603	0.8636	0.7330	0.0010
DT24	0.6818	5	22	4	0.4917	0.5000	0.4507	0.1530
DT25	0.4318	11	22	6	0.7293	0.7273	0.6939	0.4650
DT27	0.4545	6	22	4	0.6353	0.6818	0.5632	0.0060
DT28	0.3182	10	22	6	0.7531	0.8636	0.7109	0.5390
DT32	0.7045	4	22	3	0.4287	0.4091	0.3543	1.0000
DT33	0.4773	9	22	6	0.6901	0.6818	0.6499	0.4200
Mean	0.4913	7.3846	22	5.5385	0.6396	0.7273	0.5968	–

MSL, microsatellite loci; MAF, major allele frequency; Gn, number of genotypes; SS, sample size; An, number of alleles; He, expected heterozygosity; Ho, observed heterozygosity; PIC, polymorphism information content; HWE, Hardy-Weinberg equilibrium; Bonferroni adjusted *p*-value = 0.003.

## Supplementary Materials

Supplementary materials can be found at www.mdpi.com/1422-0067/17/10/1621/s1.
