# Supplementary material for: Development of 12 Microsatellite Markers in Dorcus titanus castanicolor (Motschulsky, 1861) (Lucanidae, Coleoptera) from Korea Using Next-Generation Sequencing
Source: Int J Mol Sci. 2016 Sep 23;17(10):1621. doi: 10.3390/ijms17101621 (PMC5085654; doi:10.3390/ijms17101621)
Supplement: Supplementary file 1 [file ijms-17-01621-s001.pdf]

Supplementary Materials: Development of 12 Microsatellite Markers in *Dorcus titanus castanicolor* (Motschulsky, 1861) (Lucanidae, Coleoptera) from Korea Using Next-Generation Sequencing

Tae Hwa Kang, Sang Hoon Han and Sun Jae Park

Table S1. Allele types in the Korean population of *Dorcus titanus castanicolor*.

Sample No	DT01	DT02	DT03	DT05	DT06	DT08	DT11	DT12	DT13	DT15	DT17	DT24	DT25	DT26	DT27	DT28	DT32	DT33	DT35
T151	204/204	261/261	238/241	197/200	284/316	166/181	152/180	216/218	192/210	189/203	224/227	258/258	282/291	232/268	274/278	220/226	292/298	281/281	200/204
T152	204/204	241/261	241/244	194/200	284/316	184/193	180/224	216/236	194/210	201/203	224/227	249/258	282/288	232/253	274/274	224/226	292/292	281/284	194/206
T153	204/204	241/261	241/244	197/200	284/314	166/205	180/224	218/236	192/194	187/189	206/212	246/258	282/288	232/253	274/278	220/224	292/298	269/281	200/204
T154	204/204	261/261	241/244	197/200	284/316	184/193	180/224	218/218	194/208	161/189	206/212	246/249	282/288	232/253	274/278	202/224	292/298	269/281	204/206
T155	204/204	261/261	238/241	197/200	292/294	184/205	152/180	214/216	190/192	187/189	224/227	258/258	282/282	232/253	274/278	220/226	292/292	281/287	200/204
T156	204/204	261/269	241/244	194/200	292/294	202/205	180/180	216/218	190/192	187/189	215/227	258/258	282/294	232/253	278/278	222/224	292/292	278/281	196/204
T157	204/212	261/261	241/241	194/200	284/316	202/205	180/180	214/216	190/192	187/189	224/227	258/258	282/282	232/253	274/278	202/226	292/298	281/287	200/204
T158	204/204	265/289	241/241	197/200	284/314	166/184	180/180	210/236	192/194	189/203	107/107	252/258	282/288	232/253	278/278	202/220	292/295	281/281	194/204
T159	204/212	261/261	241/241	197/200	294/314	202/205	180/180	218/236	192/194	199/201	203/203	246/249	282/288	232/253	274/278	202/220	298/298	269/281	200/206
T160	196/204	253/261	241/244	194/200	292/294	166/181	180/224	214/216	192/194	161/203	227/227	258/258	282/282	232/259	278/278	202/220	292/292	281/281	196/204
T161	204/204	241/261	241/244	194/200	292/294	184/193	180/224	216/218	192/194	187/189	224/227	249/258	288/288	232/268	266/266	202/224	292/292	281/284	204/206
T162	196/204	261/261	241/244	194/200	314/316	184/190	152/180	216/218	192/194	187/189	224/227	246/249	282/294	232/268	266/266	220/224	292/298	269/284	200/206
T163	204/204	241/261	241/244	197/200	292/294	184/193	180/228	216/236	192/194	201/203	224/227	249/258	288/291	232/253	274/278	224/224	292/292	281/281	204/206
T164	204/204	253/261	241/244	197/200	294/314	184/205	180/224	216/218	194/206	189/203	206/227	249/258	276/285	232/253	274/278	220/228	292/292	281/284	200/204
T165	204/204	261/261	241/244	197/200	294/294	184/193	152/180	218/218	192/208	189/189	206/227	258/258	282/282	223/232	266/278	202/220	298/298	281/284	194/204
T166	204/204	241/261	241/244	200/200	294/316	184/193	184/224	210/216	192/194	189/203	215/227	249/258	288/294	223/232	274/274	224/224	292/292	284/284	194/206
T168	196/204	261/261	238/241	197/200	292/294	184/205	152/180	216/216	194/208	189/189	206/227	258/258	282/291	232/253	266/282	202/224	292/292	272/272	196/200
T169	196/204	241/261	241/244	197/200	286/314	166/205	180/224	216/218	194/208	189/201	224/227	258/258	291/294	232/253	266/278	202/224	292/292	278/284	196/196
T170	204/212	241/261	244/244	194/197	284/294	190/202	180/180	234/236	192/194	199/201	215/227	258/258	285/285	232/253	274/278	220/224	292/298	281/287	194/196
T173	204/212	241/261	241/244	197/200	294/314	184/193	180/224	218/236	192/194	201/203	224/227	258/258	276/282	232/253	274/278	202/220	292/298	281/284	204/206
T174	204/204	241/261	241/244	197/200	294/314	184/193	180/180	210/218	194/210	161/203	176/191	258/258	288/291	232/253	274/278	220/224	292/298	278/281	194/206
T175	204/204	241/261	241/244	197/200	294/314	190/190	224/224	218/236	194/208	203/205	206/227	252/258	282/294	232/253	274/278	226/226	292/292	284/284	196/206

Table S2. Collection information for the Korean population of *Dorcus titanus castanicolor*.

Sn	NIBR Reg	CS	CD	Sex	Remarks
–	–	Mt. Jangtae, Jangan-dong, Seo-gu, Daejeon, Korea	July 2014	male	For NGS
T151	NIBRIN 0000732854	Mt. Jangtae, Jangan-dong, Seo-gu, Daejeon, Korea	July 2014	female	For Genotyping
T152	NIBRIN 0000732856	Mt. Jangtae, Jangan-dong, Seo-gu, Daejeon, Korea	July 2014	female	For Genotyping
T153	NIBRIN 0000732857	Mt. Jangtae, Jangan-dong, Seo-gu, Daejeon, Korea	July 2014	female	For Genotyping
T154	NIBRIN 0000732858	Mt. Jangtae, Jangan-dong, Seo-gu, Daejeon, Korea	July 2014	female	For Genotyping
T155	NIBRIN 0000732859	Mt. Jangtae, Jangan-dong, Seo-gu, Daejeon, Korea	July 2014	female	For Genotyping
T156	NIBRIN 0000732861	Mt. Jangtae, Jangan-dong, Seo-gu, Daejeon, Korea	July 2014	female	For Genotyping
T157	NIBRIN 0000732862	Mt. Jangtae, Jangan-dong, Seo-gu, Daejeon, Korea	July 2014	female	For Genotyping
T158	NIBRIN 0000732864	Mt. Jangtae, Jangan-dong, Seo-gu, Daejeon, Korea	July 2014	female	For Genotyping
T159	NIBRIN 0000732865	Mt. Jangtae, Jangan-dong, Seo-gu, Daejeon, Korea	July 2014	female	For Genotyping
T160	NIBRIN 0000732867	Mt. Jangtae, Jangan-dong, Seo-gu, Daejeon, Korea	July 2014	female	For Genotyping
T161	NIBRIN 0000732868	Mt. Jangtae, Jangan-dong, Seo-gu, Daejeon, Korea	July 2014	female	For Genotyping
T162	NIBRIN 0000732869	Mt. Jangtae, Jangan-dong, Seo-gu, Daejeon, Korea	July 2014	female	For Genotyping
T163	NIBRIN 0000732870	Mt. Jangtae, Jangan-dong, Seo-gu, Daejeon, Korea	July 2014	male	For Genotyping

Table S2. *Cont.*

Sn	NIBR Reg	CS	CD	Sex	Remarks
T164	NIBRIN 0000732872	Mt. Jangtae, Jangan-dong, Seo-gu, Daejeon, Korea	July 2014	male	For Genotyping
T165	NIBRIN 0000732873	Mt. Jangtae, Jangan-dong, Seo-gu, Daejeon, Korea	July 2014	male	For Genotyping
T166	NIBRIN 0000732874	Mt. Jangtae, Jangan-dong, Seo-gu, Daejeon, Korea	July 2014	male	For Genotyping
T168	NIBRIN 0000732875	Mt. Jangtae, Jangan-dong, Seo-gu, Daejeon, Korea	July 2014	male	For Genotyping
T169	NIBRIN 0000732877	Mt. Jangtae, Jangan-dong, Seo-gu, Daejeon, Korea	July 2014	male	For Genotyping
T170	NIBRIN 0000732878	Mt. Jangtae, Jangan-dong, Seo-gu, Daejeon, Korea	July 2014	male	For Genotyping
T173	NIBRIN 0000732879	Mt. Jangtae, Jangan-dong, Seo-gu, Daejeon, Korea	July 2014	male	For Genotyping
T174	NIBRIN 0000732880	Mt. Jangtae, Jangan-dong, Seo-gu, Daejeon, Korea	July 2014	male	For Genotyping
T175	NIBRIN 0000732882	Mt. Jangtae, Jangan-dong, Seo-gu, Daejeon, Korea	July 2014	male	For Genotyping

Sn, Sample No; NIBR Reg, Registeration ID of National Institute of Biological Resources; CS, Collection Site; CD, Collection Date; NGS, Next Generation Sequencing.
